# Power Control during Remote Laser Welding Using a Convolutional Neural Network

**DOI:** 10.3390/s20226658

**Published:** 2020-11-20

**Authors:** Alex Božič, Matjaž Kos, Matija Jezeršek

**Affiliations:** Laboratory for Laser Techniques, Faculty of Mechanical Engineering, University of Ljubljana, Aškerčeva cesta 6, 1000 Ljubljana, Slovenia; alex.bozic21@gmail.com (A.B.); matija.jezersek@fs.uni-lj.si (M.J.)

**Keywords:** convolutional neural network, remote laser welding, laser-power control, triangulation feedback

## Abstract

The increase in complex workpieces with changing geometries demands advanced control algorithms in order to achieve stable welding regimes. Usually, many experiments are required to identify and confirm the correct welding parameters. We present a method for controlling laser power in a remote laser welding system with a convolutional neural network (CNN) via a PID controller, based on optical triangulation feedback. AISI 304 metal sheets with a cumulative thickness of 1.5 mm were used. A total accuracy of 94% was achieved for CNN models on the test datasets. The rise time of the controller to achieve full penetration was less than 1.0 s from the start of welding. The Gradient-weighted Class Activation Mapping (Grad-CAM) method was used to further understand the decision making of the model. It was determined that the CNN focuses mainly on the area of the interaction zone and can act accordingly if this interaction zone changes in size. Based on additional testing, we proposed improvements to increase overall controller performance and response time by implementing a feed-forward approach at the beginning of welding.

## 1. Introduction

Remote laser welding is a fast, complex process that achieves fast welding speeds and greater penetration depths at higher accuracy over a larger working area. It is especially used in the automotive, electronics, and appliance industry [1,2,3,4]. Issues occur when these systems are used for applications where mass customization and dissimilar materials are present, as it is time consuming and inaccurate to train them for different welding paths and process parameters [5,6,7] in addition to achieving good weld quality [8,9,10] and process traceability.

The first challenge in laser welding applications is to accurately deliver the laser beam to the weld location. This is done with off-line simulations [11] or in-line with an optical method [12,13,14,15]. Secondly, welding processes can be predicted to some extent by FEM analyses [16], numeric simulations [17], experiment response surface methodology [18,19], or knowledge from previous studies [20,21], and can often lack the acquired transferability to other welding processes due to different welding conditions (type of material, laser characteristics). However, the key issue is in comprehending the welding process itself, e.g., melt pool dynamics [22,23], plasma dynamics [24,25], or the effect of material properties [26], with which new welding parameters for various material combinations and joint configurations can be established.

Recent advances in machine learning allows connection and merging of different sensor signals, e.g., multiple cameras and photodiodes [27], or addition of post-welding quality analysis [28]. This provides a new way to develop algorithms to identify and monitor key welding parameters and to control the process itself. Among other machine learning algorithms, convolutional neural networks (CNN) have a great advantage as they can also handle 2D images of the process [29,30,31,32] to control welding parameters. They are efficient, highly adaptable, and can be easily trained, which is a benefit when considering scalability options where numerous input parameters are available, as in laser welding applications. Zhang et al. [33] developed a monitoring system for the penetration status of conventional laser welding consisting of a coaxial fast camera. This enabled them to correctly determine if partial, moderate, full, or excessive penetration occurred with 95% accuracy from acquired images and claimed it outperformed other vision-based monitoring applications. However, the system is limited in the field of view of the coaxially placed camera and in focus monitoring of the welding head.

The objective of this paper was to demonstrate the applicability of a CNN in a remote laser welding system in order to control the laser power during lap welding. The method is based on an optical triangulation loop, which can be simultaneously used for welding process classification by the CNN while monitoring the working area and for in-line 3D seam tracking, recently presented in [34]. The camera is mounted on a scanning head, retaining the ability to monitor the scanning head’s larger working area. The CNN is designed as a modular system, which classifies the current state of the remote welding process and controls the power output of the laser via the PID controller to achieve a desired output of the process. Three distinct welding states or categories were defined: (i) the energy input to the workpiece is too small (i.e., only partial penetration is present); (ii) the energy input is sufficient (i.e., full penetration is present); or (iii) the energy input is over-exceeded (unnecessary increase of the weld width and heat-affected zone). A PID controller was then developed to change the absolute laser power based on the classifications of the CNN in real-time. The desired output of the controller was set to achieve full penetration on the specimen. Welding experiments were carried out on AISI 304 steel sheets to demonstrate the responsiveness, stability, and overall accuracy of the controller during remote laser welding. The main idea of this kind of control algorithm is that the needed input laser power is unknown in advance. The PID controller changes the absolute laser power based on the classifications of the CNN. Experiments demonstrate that the desired full penetration can be achieved with the developed controller.

This paper is organized as follows: Section 2 presents the remote laser welding system, the CNN architecture, and the method of preparing the dataset for training the network. A description of the experimental method and classification process is given. Section 3 provides the results and discussion, where the performance of the CNN classification is evaluated and an analysis on the decision making is done. The performance of the network is tested on actual welding. A conclusion is given in Section 4.

## 2. Materials and Methods

### 2.1. Remote Laser Welding System

**Figure 1:**
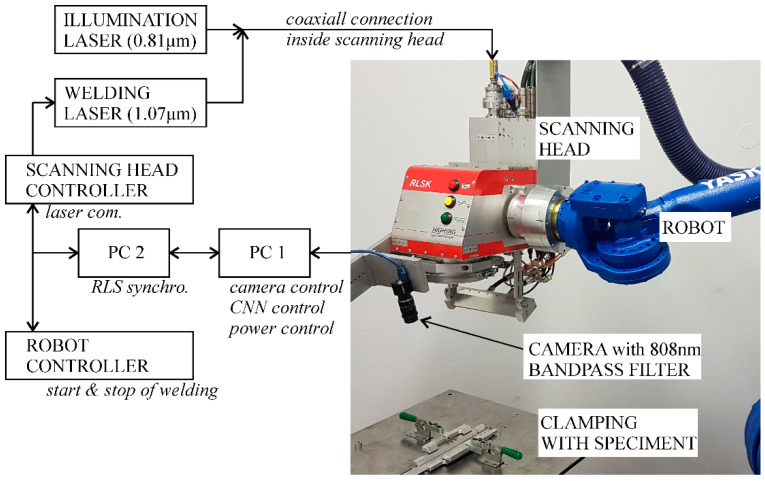
Experimental system and setup.

The system for remote laser welding is shown in Figure 1. It consists of an industrial robot (Yaskawa GP50, Yaskawa Slovenija d.o.o., Ribnica, Slovenia ), a scanning head (HighYag RLSK, II-VI Incorporated, Weiterstadt, Germany, with a working area of 200 mm × 300 mm × 200 mm in the x, y, and z directions, respectively), and a fiber laser (IPG, YRL-400-AC, IPG Photonics, Burbach, Germany, wavelength of 1.07 µm; maximum power of 400 W). An illumination laser (Fotona XD-2, Fotona d.o.o., Ljubljana, Slovenia, 810 nm ± 10 nm) coaxially connected to the fiber laser’s beam path was used in order to equalize the brightness of the interaction zone and the surrounding area. A console-attached camera (Flea3, FLIR, Frankfurt am Main, Germany, model FL3-U3-13Y3M-C) with a band-pass filter (FBH810-10, Thorlabs GmbH, Bergkirchen, Germany, central wavelength 810 nm, FWHM = 10 nm) was used to monitor the interaction zone between the laser and the workpiece. A resolution of 1280 × 1024 with a frame rate of 80 FPS was used for the experiments. The triangulation angle between the camera and the scanning head was 15° and the resolution of the optics was 0.1 mm. The calculated beam diameter in the focus of the system was 0.032 mm. All experiments were carried out in focus.

Two computers were used: The first computer (Acer, Intel Core i7 @ 2.2 GHz, 16 GB RAM and NVidia GeForce GTX 1060 6GB VRAM) acquired images from the camera and determined the laser power via the developed controller with software developed in Python. The second computer was used for the interface and to synchronize the start and end of the welding between the robot, scanning head, and the laser with the LabView software.

### 2.2. Methodology of CNN Model Creation

When designing a laser welding process, one has to firstly specify the desired welding result to be achieved (in terms of penetration depth and weld width) and at which welding speed the process should be conducted. Typical laser welding applications usually determine the desired penetration depth (partial penetration or full penetration) and load‑bearing criteria of the weld (which mostly correlates with the weld width). This research is focused only on the first part, i.e., to control the penetration depth with a CNN through laser‑power control.

**Figure 2:**
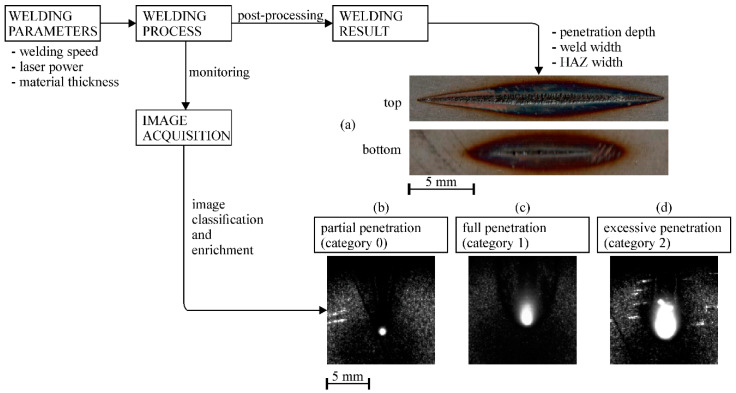
Block diagram for welding result classification and image preparation for the convolutional neural network (CNN). (**a**) Top and bottom side of an example lap welding of the 1.5 mm steel plate made with linear power change at 50 cm/min is shown. Acquired representative images at (**b**–**d**) depicting different penetration states are extracted from the process.

We concluded that three welding results are most common and can be easily distinguished: partial penetration, full penetration, and excessive penetration (see Figure 2). These results are primarily determined by a combination of laser power and welding speed used, and secondly by material type and material thickness. A combination of different welding parameters can produce similar welding results. Normally, this knowledge is obtained through experimentation or simulations. In order to train the CNN model to recognize which welding-parameter combinations produce these results, typical images depicting different welding states for a given welding application must first be acquired. Different welding states can be obtained while welding at a specific welding speed and simultaneously changing the laser power. Figure 2 shows an example of such an experiment where a lap weld of 1.5 mm thick steel plates was made at 50 cm/min by linearly changing the laser power from minimum to maximum and back to minimum power. Welding was monitored with the attached camera. From the top and bottom side of the made weld (see Figure 2a), we can depict the start and the end of penetration. This helps us sort the acquired images into selected penetration states, from partial penetration to excessive penetration (as shown in Figure 2b–d).

The power control algorithm was trained for laser lap welding of two commercially available stainless steel plates (AISI 304), with a cumulative thickness of 1.5 mm (0.5 mm plate on 1.0 mm plate) for welding speeds of 25, 50, 75, and 100 cm/min where these values represent the expected range of welding results (from partial to excessive penetration) when using a laser source of 400 W. At lower welding speeds (below 25 cm/min), heat conduction and accumulation in the surrounding material are profound, resulting in faster excessive penetration. In addition, the heat accumulation makes it harder to distinguish the boundary between sufficient and excessive energy input. On the contrary, higher welding speeds (above 100 cm/min) were omitted as the equipment was unable to output enough power to achieve excessive penetration, which is needed to correctly train the network. Experiments for all investigated welding speeds were carried out in focus. Because we wanted to establish only the boundaries between penetration states, linear welding was performed. Sections of made welds were divided into the specified categories and grouped with the corresponding images and laser power. Overall, four independent networks were trained for each speed separately.

**Figure 3:**
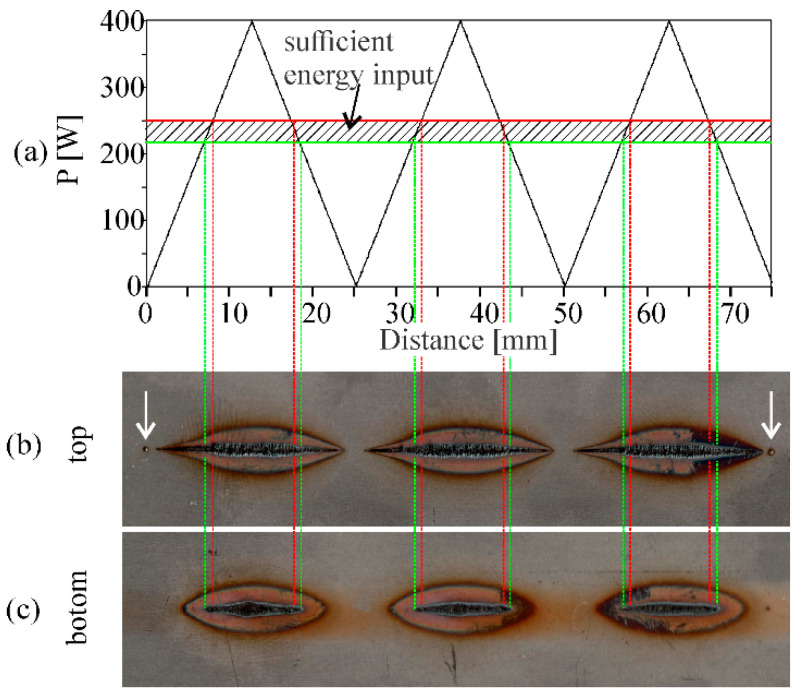
Experimental procedure to determine the power borders between too little, sufficient, and exceeding penetration for a welding speed of 25 cm/min. (**a**) Power change during welding; (**b**,**c**) top and bottom side of the weld. Arrows pointing to reference marks at the beginning and ending of the weld in (**b**) were used for synchronization purposes. Vertical lines show the averaged power borders between categories. The graphs and the weld are aligned.

Power borders between different categories were established using the changing laser power method, from 0 W to 400 W and back to 0 W as shown in Figure 3a. In the figure, an example welding at 25 cm/min is shown. Reference marks are placed at the beginning and ending of the welding (spot welds marked with arrows, seen in Figure 3b). They were used to properly connect the acquired images and laser power with the corresponding weld result. By measuring the laser power at which full penetration occurred on the back side of the workpiece (as seen in Figure 3c), the boundary between too little energy input (category 0) and sufficient energy input (category 1) was established. This power presents the lower power border that the CNN should obtain in order to achieve full penetration. A 40 W higher value, which presents a 10% change of overall laser power, was determined to be the boundary between sufficient energy input (category 1) and exceeding energy input (category 2). This presents the upper power border. The process was repeated and average power borders between different categories were established for investigated welding speeds (see Table 1).

Because the scanning head can move the laser beam, the interaction zone was detected automatically using the algorithms presented in a previous work [34]. These algorithms consist of five steps, namely, robust detection of the illuminated area, filtering with a Gaussian kernel to emphasize the interaction area in the illuminated area, applying an overall image threshold equal to 95% value of the maximum pixel intensity, checking previous keyhole locations, eliminating regions corresponding to spatter, and calculating the centroid of the remaining region. Image enrichment in terms of rotating the image around the center of the detected interaction zone was done in order to compensate for different welding directions. A smaller image with a resolution of 128 × 128 was then extracted.

### 2.3. CNN Architecture

**Figure 4:**
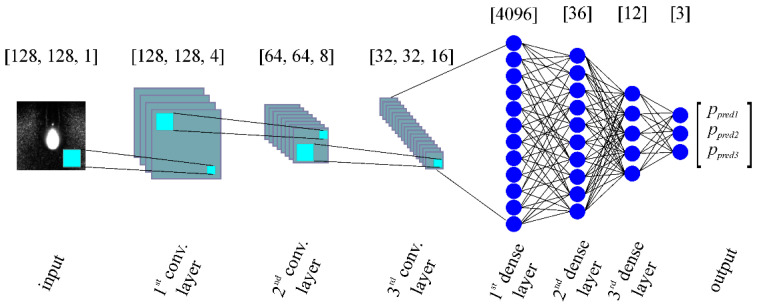
Schematic of the architecture used for the CNNs. The output gives the probability to which category an input image corresponds to.

Figure 4 shows the architecture that was used to create the CNN network. The input of the CNN was an 8-bit grayscale image obtained from the experiments described in Section 2.2. The architecture of CNN was implemented in Keras [35]. Three convolutional layers were used to extract the features from the input image. Each layer contains the following steps: (i) convolution, (ii) batch normalization, (iii) activation function, and (iv) pooling.

The parameters for creating the neural network were selected with the help of general guidelines [36,37] with responsiveness as the main focus. Additional empirical tests were carried out to obtain preferable results. Therefore, the size of the convolutional kernel was set to 3 × 3 globally, which reduced the receptive field and made it capable of detecting minute details. Filter sizes between convolutional layers increased by a factor of 2 and 4, respectively, according to the previous filter’s size. Default values were used for batch normalization. The pooling layer used had a window size of 2 × 2 and was activated with a max-value operation. Dropout with a factor of *p* = 0.45 (determined based on more attempts to build a successful model) was implemented to decrease the chances of overfitting. The output of the model was acquired with softmax activation; thus, the output value is a vector of a probability distribution of a list of potential outcomes according to the CNN model. Given that the categories are of ordinal type, this information was used in such a way that the output vector was considered as if it was the actual probability distribution.

### 2.4. Dataset Preparation

Roughly 3000 images were used for each speed and category in order to eliminate bias that an imbalance of the dataset can inflict. Each image batch was further divided into three sets: learning, validation, and test set. Because of a relatively high acquisition speed compared to the welding speed, two consecutive images had a significant correlation. Therefore, we left out approximately 50 images between two subsets to reduce their correlation. Moreover, to further distinguish between the categories, images acquired from the 5% around the power borders were omitted.

Furthermore, because of the use of an additional illumination laser, different types of surface irregularities, such as scratches, dents, etc., that occur on an overexposed area on the camera were seen on the workpiece. In order to deter the CNN from relying on these irregularities, we randomly added some type of surface defect to approximately 30% of all acquired images in different orientations.

### 2.5. PID Controller Algorithm

The main idea behind this controller is that it adapts the input power on its own accord, thus the input power value is unknown to the welder. The constructed CNN has three outputs that correspond with the probability of which category an acquired image belongs to. Thus, the error value for a discrete time *e_i_* is calculated using

(1) $$e_{i}=C_{norm}\cdot p_{pred},$$

where *C_norm_* denotes the discrete values of the categories normalized around zero, [1, 0, −1]; *p_pred_* stands for the probability an image corresponds to the defined categories. This normalization allowed us to represent our desired controlled state (sufficient laser power—category 1) as an error equal to 0, which means no action is needed. Too little or exceeding energy input means that we have an error, the size of which is equal to the probability that an acquired image corresponds to each category. This simplifies our equation to

(2) $$e_{i}=\left(+1,\;0,-1\right)\;\cdot \left(p_{pred1},\;p_{pred2},\;p_{pred3}\right)=p_{pred1}\;-\;p_{pred3}.$$

A classical PID controller was used to control the necessary change in laser power using the error value:

(3) $$\Delta P_{i}=K_{p}\cdot e_{i}+K_{i}\cdot \overset{n}{\underset{i=0}{\sum }}e_{i}+K_{d}\cdot \frac{e_{i}-e_{i-1}}{dt}.$$

The coefficients of the controller were experimentally determined using the Ziegler–Nichols method for each welding speed separately when testing the controller stability.

### 2.6. CNN Validation and Power Control Experimentation

Four CNN models were made with permutations of the dataset and the best model was used to test its accuracy on the test set and later during actual welding. The actual welding was performed by changing laser power from 0 W to 400 W.

To understand the CNN’s decision making process and to understand why errors in the decision making actually occur, the Gradient-weighted Class Activation Mapping (Grad-CAM) technique was used [38]. This technique uses the gradient information flowing into the last convolutional layer of the CNN to understand the importance of each neuron for a decision of interest. It uses an image and a class of interest as an input to the CNN to obtain a raw score for the category. A coarse localized heat map is then created to gain more insight into which regions of the image contain important features for a particular decision.

The performance of the PID controller (overshoot, rise time, and settling time) was tested with and without disturbance of the laser welding process. The disturbance was implemented as an artificial change of the laser power to minimum or maximum power for 0.5 s. The tests were carried out for all investigated welding speeds. The weld length was 50 mm with a starting laser power of 0 W. The PID coefficients determined for stable operation for each speed are shown in Table 2. In the end, two methods were tested to achieve a faster and more stable response: changing the camera frame rate and welding with a feed-forward approach at the beginning.

## 3. Results and Discussion

**Figure 5:**
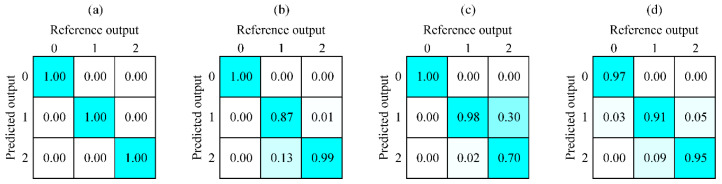
Confusion matrix for each speed. Every column represents correct classification with 100% probability. The ordinate shows the predicted output from the CNN model: 0—too little energy input, 1—sufficient energy input, 2—exceeding energy input. (**a**) Welding speed of 25 cm/min; (**b**) welding speed of 50 cm/min; (**c**) welding speed of 75 cm/min; (**d**) welding speed of 100 cm/min.

All CNN models became almost stagnant at 200 epochs; thus, the learning phases were concluded at this stage. To get a general indication of the CNN model’s performance, the model was tested on the test data. Given the ordinal classes, it is important that misclassification occurs only between adjacent classes, e.g., an image from category 0 should not be predicted as category 2. In Figure 5, we can see four different confusion matrices, one for each speed. All confusion matrices indicated good performance of the model, as a total accuracy of 94% was achieved. However, most of the misclassifications occurred between categories 1 and 2, particularly for a welding speed of 75 cm/min (Figure 5c). As test data images are part of the same weld as the training and evaluation dataset, some correlation between images is expected. In addition, the difference between categories 1 and 2 for greater welding speeds is harder to determine, as the energy input to the surrounding of the interaction zone is lower, i.e., the camera cannot observe the material change because of the higher laser power as the material itself does not respond quickly enough. That is why these results are only general indications and not the actual representations of the model’s performance. To get relevant results, the model was also tested on actual welding.

**Figure 6:**
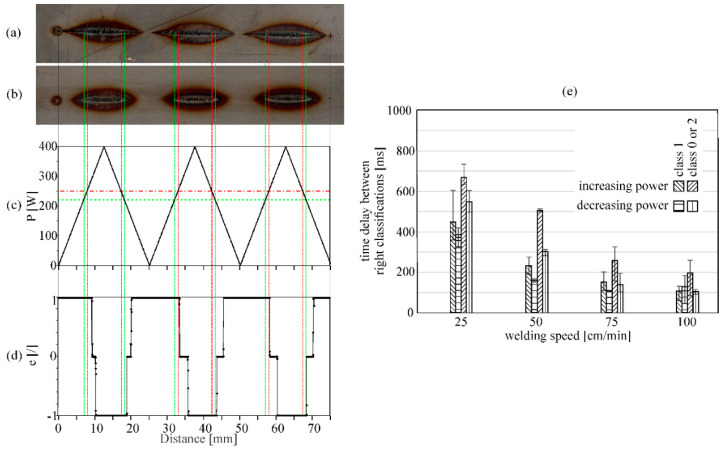
Example of CNN model response to actual welding at 25 cm/min. (**a**) Top of workpiece; (**b**) bottom of workpiece; (**c**) power change during welding; (**d**) error value given from the CNN model; (**e**) analysis of classification delay for different welding speeds. Horizontal lines in graph (**c**) show the power border between too little energy input (green) and exceeding (red) energy input defined at the learning step. Vertical lines indicate the beginning and end of welding (black) and when the CNN model should change the output. The graphs and the weld are aligned.

Figure 6 shows an example response of the CNN model to an actual welding process with changing laser power at a welding speed of 25 cm/min. From graph (d), we notice that the actual classification from the model lags behind the welding process. Sufficient energy input classification is always obtained after the welding process crosses the power border for exceeding energy input (slope from 0 to 400 W) or too little energy input (slope from 400 to 0 W). We assume that this can be explained by the slower heating rate of the material. As the camera monitors the welding process in the NIR field (810 nm), some delay from the actual response was expected as heat emissions are also detected in this spectrum. To increase the accuracy and get a better estimation of the actual size of the interaction zone, a different illumination source in the visible spectrum should be used, e.g., high power monochromatic LED. This would enable us to monitor the actual size of the melt poll or even the keyhole. Furthermore, the detection of workpiece features, such as edges, would still be visible and could enable combined control of laser power and position.

Figure 6e shows just how much delay is present between the right classifications for different welding speeds. When decreasing laser power (from 400 to 0 W), the time offset between classifications decreases. This is due to the accumulated heat in the material. Furthermore, because of this accumulated heat, sufficient energy input is later obtained faster during the increase of the laser power. From Figure 6e, we can see that the time delay shortens with increasing welding speed from an average of 0.5 s to less than 0.2 s, which is due to the fact that the material is not heated to such an extent.

We consider that this kind of material behavior is normal and can be a ground to develop dual control algorithms—one determining the steady-state process (what kind of interaction must be achieved to get the desired penetration depth) and another to monitor and control transient states that occur during welding, e.g., how does the area of the interaction zone change when full penetration is achieved or, moreover, how does the development of a keyhole affect a simple controller due to the non-linear change in penetration depth.

**Figure 7:**
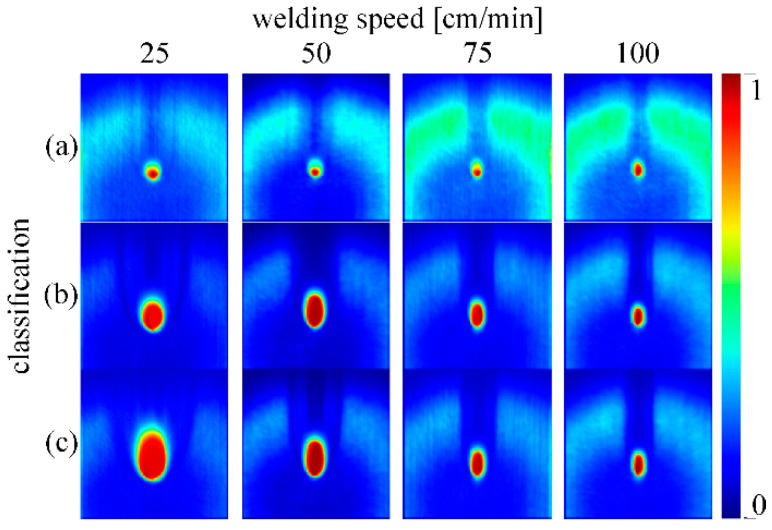
Result of the Gradient-weighted Class Activation Mapping (Grad-CAM) method at the output of the first convolutional layer. Columns present different welding speeds from 25 cm/min to 100 cm/min. Rows present categories for too little (**a**), sufficient (**b**), and exceeding (**c**) energy input. Values are color-coded, where red means that the pixel has an impact on decision making and blue means that the pixel has no impact.

To determine which image features the CNN model concentrates on, a coarse localization map with the Grad-CAM method was used. Figure 7 shows the results of the Grad-CAM method for every welding speed (columns) and classifications for different penetration states (rows). It can be concluded that the most significant part of the acquired image is the interaction zone area. This was expected as the interaction zone is the only part of the image that changes during welding. What is important is the relationship of how this interaction zone changes with changing welding parameters, e.g., different welding speeds. We notice that too little energy input has a significantly smaller interaction zone and that the illumination laser is easily seen (a). Greater laser power correlates with a larger melt pool and greater temperature rise of the material, which can be monitored with a camera. For the case of sufficient (b) and exceeding energy input (c), the difference is seen only in the size of the interaction zone. We can interpret that this also relates to the accuracy reduction of the model at greater welding speeds, as the difference in the interaction zone is negligible. We assume the reason for this lies in the fact that the power border between these two categories cannot be uniquely determined as the laser power and system specifications do not allow us to achieve the required intensities that are needed for this situation.

**Figure 8:**
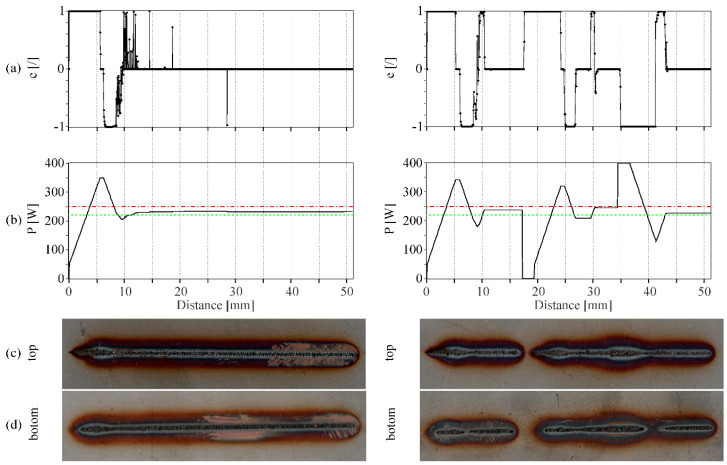
Example of CNN model classification and control of laser power of a welding process at 25 cm/min. The left-hand side depicts the welding process with no disturbances while the right-hand side depicts the welding process with two disturbances of length 0.5 s or 2.1 mm. (**a**) Error value given from the CNN model; (**b**) laser power output given from the PID controller; (**c**) top side of the weld; (**d**) bottom side of the weld. Horizontal lines in graph (**b**) show the power border between too little (green) and exceeding (red) energy inputs defined at the learning step. The graphs and the welds are aligned.

Because of the nature of the process and thus the lagging of category classification of the CNN model, an induced overshoot can be expected. This is shown in Figure 8, where we can see the process of power control for a welding speed of 25 cm/min for a remote laser welding process with the developed CNN model. The left-hand side of the figure presents the control without artificial power disturbances and the right-hand side presents the control with two implemented power disturbances of 0 W and 400 W for a duration of 0.5 s (or 2.1 mm). Even though overshoots are present in all cases, sufficient energy input classification is achieved. Brief misclassification that occurs during welding is due to plume blowouts and spatter debris, which can cause a short enlargement of the interaction zone. Nonetheless, the power change during these situations is minuscule from which we can conclude that the added surface defects in the training part of the model are correct and necessary.

**Figure 9:**
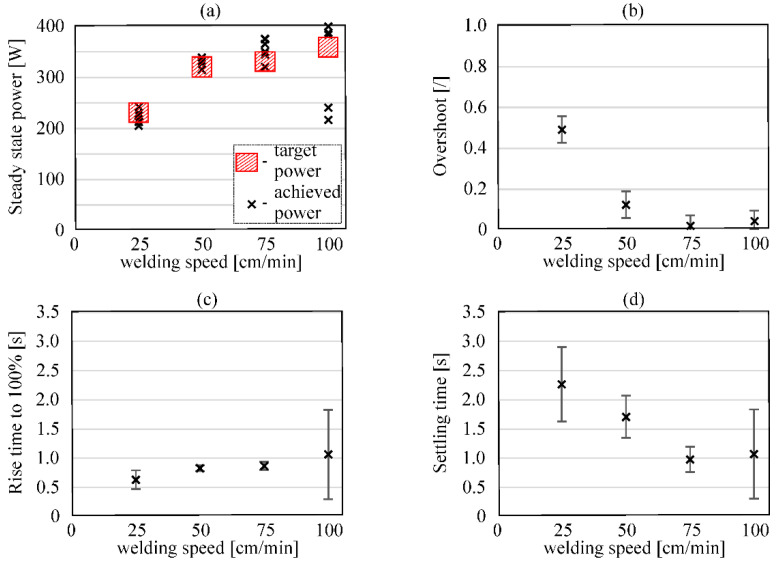
Performance of the developed power control model for investigated welding speeds. (**a**) Achieved steady-state power with determined target power borders for sufficient energy input; (**b**) overshoot; (**c**) rise time; (**d**) settling time.

The performance of the developed power control model for all considered welding speeds is seen in Figure 9. In the figure, graph (a) presents the achieved steady-state power for each welding speed with the power borders that depict the category for sufficient energy input determined at the learning stage of the controller. On graphs (b, c, and d), the overshoot, rise time to 100%, and settling time, respectively, are shown. From the experiments, we can see a poor performance for the welding speed of 100 cm/min. This is due to the previously stated fact examined in Figure 7—the difference between sufficient energy input and exceeding energy input cannot be uniquely determined because of the welding system limitations. This is further seen as the error between the steady-state power achieved after settling and the determined target power for sufficient energy input (Figure 9a). However, for welding speeds of 25, 50, and 75 cm/min, the developed model exhibits good performance, as most of the experiments fall inside the target power. Graph (c) shows an average rise time of less than 1 s and quicker rise times for a lower welding speed as the power needed for sufficient energy input is much lower. Settling times are longer and are present because of the overshoot. Little overshoot, as seen for the welding speed of 75 cm/min, produces less difference between rise times and settling time.

The system underperforms at higher welding speeds and has a rather high overshoot and long settling times at lower welding speeds. The first case is due to the system’s inability to distinguish between categories and the second case is due to the energy buildup in the material (Figure 6e), which results in overheating and subsequent power oscillation. In order to get a good performance, the developed model should be used for welding speeds, where the categories can still be differentiated and there is little accumulated heat, as seen for welding speeds of 50 and 75 cm/min.

**Figure 10:**
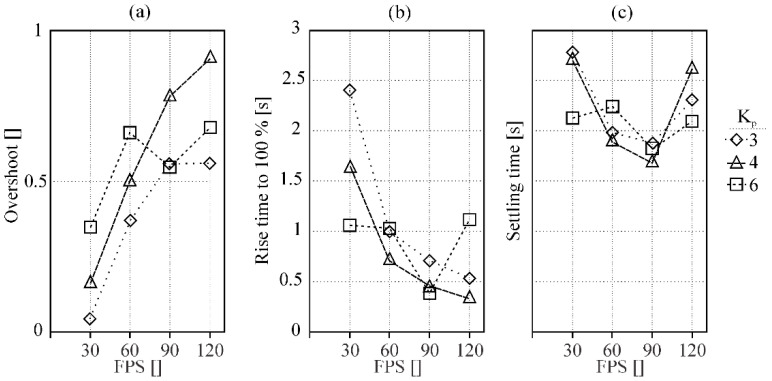
Dependency of controller overshoot (**a**), rise time (**b**), and settling time (**c**) for different camera frame rates and only proportional parameters for a welding speed of 25 cm/min.

A possible solution to improve the response time is to increase the acquisition frame rate of the camera and consequently the frequency of determining and setting a new power. Figure 10 depicts how the properties of the controller (overshoot (a); rise time (b); settling time (c)) for a welding speed of 25 cm/min are affected by changing the camera frame rate (FPS) and proportional parameter of the controller.

With greater FPS (Figure 10b), we get a better response time but due to the previously mentioned material response, the settling time does not shorten as more instability is delivered as a result of greater overshoot. Furthermore, even changing other PID control coefficients would not have any significant impact on overall controller performance as the CNN classification would still have a delay.

**Figure 11:**
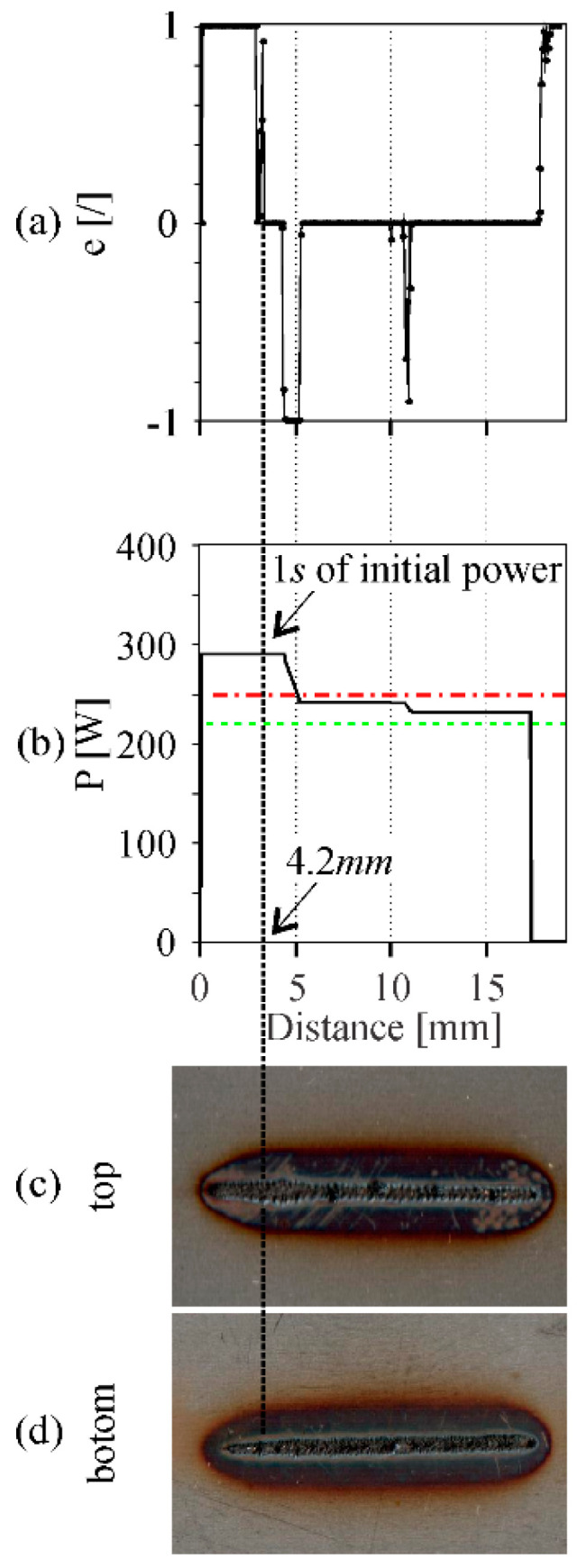
Overshoot elimination with a feed-forward approach, where an initial power equal to 115% of *P_H_* for 1.0 s is used to preheat the workpiece. (**a**) Error value given from the CNN model; (**b**) laser power output given from the PID controller; (**c**) top side of the weld; (**d**) bottom side of the weld. Welding speed of 25 cm/min was used. The graphs and the weld are aligned.

To solve the problem of overshoot at the beginning of the welding, a feed-forward approach can be used. An initial power that is slightly higher than the predetermined power border for exceeding energy input can be used in order to heat the material and reduce the CNN delay classification. An example of such an approach for a welding speed of 25 cm/min is shown in Figure 11. The initial feed-forward power was 115% of the upper power border and it lasted for 1 s. As seen in Figure 11a, even after the elapsed second, the response of the workpiece shows that the energy input is sufficient and corresponds to the constant weld width at the top of the workpiece, as seen in Figure 11c. Only later, when the weld width increases, the CNN changes the classification and lowers the laser power, falling into the predetermined power borders. This shows that the CNN is able to detect the changes in the size of the interaction zone when overheating of the workpiece occurs and can correct the laser power accordingly. However, we assume a higher FPS is still needed in order to achieve even faster response times if a disturbance occurs.

## 4. Conclusions

A novel method for laser-power control during remote laser welding based on a convolutional neural network was proposed and implemented. The method is based on acquiring images of the welding process with a triangulation camera, processing them with the developed convolutional neural network, and using the results in a PID control loop. The CNN classifies the welding process with an accuracy of 94% into three possible categories: partial, full, or exceeding penetration.

The developed power control model shows good performance, especially for a welding speed of 75 cm/min, as little overshoot is present and settling times are less than 1 s. A further reduction in settling time of at least 30% and minimal power overshoot can be achieved with a feed‑forward approach. For greater welding speeds, the model underperforms due to the system’s limitation to achieve required laser power levels.

To improve the power control method, the CNN should not only be taught on images and what state of penetration they represent, but also on the type of material and laser intensities being used to make it more general and easily scalable.

## Figures and Tables

**Table 1:** Average power borders between categories for each tested welding speed.

Welding Speed [cm/min]	Lower Power Border *P_L_* [W]	Upper Power Border *P_H_* [W]
25	210	250
50	300	340
75	310	350
100	340	380

**Table 2:** Average power borders between categories for each tested welding speed.

Speed [cm/min]	K_p_	K_i_	K_d_
25	4	0.01	0
50	2.5	0.0025	0.0001
75	4	0	0
100	2.5	0.0025	0.0001

## References

[1] Fysikopoulos A., Pastras G., Stavridis J., Stavropoulos P., Chryssolouris G. (2016). On the Performance Evaluation of Remote Laser Welding Process: An Automotive Case Study. Procedia CIRP.

[2] Ceglarek D., Colledani M., Váncza J., Kim D.-Y., Marine C., Kogel-Hollacher M., Mistry A., Bolognese L. (2015). Rapid deployment of remote laser welding processes in automotive assembly systems. CIRP Ann..

[3] Pires J.N., Loureiro A., Bolmsjö G. (2015). Welding Robots: Technology, System Issues and Applications.

[4] Buehrle J., Bea M., Brockmann R., SAE-China, FISITA (2013). Laser Remote Process Technology on Automotive Manufacture. Proceedings of the FISITA 2012 World Automotive Congress.

[5] Um J., Stroud I.A. (2017). Design guidelines for remote laser welding in automotive assembly lines. Int. J. Adv. Manuf. Technol..

[6] Bednar S., Modrak V. (2014). Mass customization and its impact on Assembly process´ complexity. Int. J. Qual. Res..

[7] Hu S.J., Ko J., Weyand L., ElMaraghy H.A., Lien T.K., Koren Y., Bley H., Chryssolouris G., Nasr N., Shpitalni M. (2011). Assembly system design and operations for product variety. CIRP Ann. Manuf. Technol..

[8] Stavridis J., Papacharalampopoulos A., Stavropoulos P. (2018). Quality assessment in laser welding: A critical review. Int. J. Adv. Manuf. Technol..

[9] Wang T., Chen J., Gao X., Li W. (2017). Quality Monitoring for Laser Welding Based on High-Speed Photography and Support Vector Machine. Appl. Sci..

[10] Poprawe R., König W. (2001). Modeling, monitoring and control in high quality laser cutting. CIRP Ann.-Manuf. Technol..

[11] Hatwig J., Reinhart G., Zaeh M.F. (2010). Automated task planning for industrial robots and laser scanners for remote laser beam welding and cutting. Prod. Eng..

[12] Muhammad J., Altun H., Abo-Serie E. (2016). Welding seam profiling techniques based on active vision sensing for intelligent robotic welding. Int. J. Adv. Manuf. Technol..

[13] Nele L., Sarno E., Keshari A. (2013). An image acquisition system for real-time seam tracking. Int. J. Adv. Manuf. Technol..

[14] Gu W.P., Xiong Z.Y., Wan W. (2013). Autonomous seam acquisition and tracking system for multi-pass welding based on vision sensor. Int. J. Adv. Manuf. Technol..

[15] Huang Y., Xiao Y., Wang P., Li M. (2012). A seam-tracking laser welding platform with 3D and 2D visual information fusion vision sensor system. Int. J. Adv. Manuf. Technol..

[16] Huang H., Wang J., Li L., Ma N. (2016). Prediction of laser welding induced deformation in thin sheets by efficient numerical modeling. J. Mater. Process. Technol..

[17] Wang R., Lei Y., Shi Y. (2011). Numerical simulation of transient temperature field during laser keyhole welding of 304 stainless steel sheet. Opt. Laser Technol..

[18] Bandyopadhyay K., Panda S.K., Saha P. (2016). Optimization of Fiber Laser Welding of DP980 Steels Using RSM to Improve Weld Properties for Formability. J. Mater. Eng. Perform..

[19] Altarazi S., Hijazi L., Kaiser E. Process parameters optimization for multiple-inputs-multiple-outputs pulsed green laser welding via response surface methodology. Proceedings of the 2016 IEEE International Conference on Industrial Engineering and Engineering Management (IEEM).

[20] Jacques L., Ouafi A.E. (2017). Experimental Investigation of Laser Welding Process in Butt-Joint Configurations. World J. Eng. Technol..

[21] Kannatey-Asibu E. (2009). Principles of Laser Materials Processing.

[22] Schaefer M., Kessler S., Fetzer F., Graf T. (2017). Influence of the focal position on the melt flow during laser welding of steel. J. Laser Appl..

[23] Eriksson I., Powell J., Kaplan A.F.H. (2013). Melt behavior on the keyhole front during high speed laser welding. Opt. Lasers Eng..

[24] Pang S., Chen X., Shao X., Gong S., Xiao J. (2016). Dynamics of vapor plume in transient keyhole during laser welding of stainless steel: Local evaporation, plume swing and gas entrapment into porosity. Opt. Lasers Eng..

[25] Miyazaki Y., Katayama S. (2015). Influence of laser-induced plume on penetration in laser welding. Weld. Int..

[26] Assuncao E., Williams S. (2014). Effect of material properties on the laser welding mode limits. J. Laser Appl..

[27] Zhang Y., You D., Gao X., Wang C., Li Y., Gao P.P. (2019). Real-time monitoring of high-power disk laser welding statuses based on deep learning framework. J. Intell. Manuf..

[28] Kurniadi K.A., Ryu K., Kim D. (2014). Real-time parameter adjustment and fault detection of remote laser welding by using ANN. Int. J. Precis. Eng. Manuf..

[29] Günther J., Pilarski P.M., Helfrich G., Shen H., Diepold K. (2016). Intelligent laser welding through representation, prediction, and control learning: An architecture with deep neural networks and reinforcement learning. Mechatronics.

[30] Zhang Y., Gao X., Katayama S. (2015). Weld appearance prediction with BP neural network improved by genetic algorithm during disk laser welding. J. Manuf. Syst..

[31] Gonzalez-Val C., Pallas A., Panadeiro V., Rodriguez A. (2019). A convolutional approach to quality monitoring for laser manufacturing. J. Intell. Manuf..

[32] Xie Y., Heath D.J., Grant-Jacob J.A., Mackay B.S., McDonnell M.D.T., Praeger M., Eason R.W., Mills B. (2019). Deep learning for the monitoring and process control of femtosecond laser machining. J. Phys. Photonics.

[33] Zhang Z., Li B., Zhang W., Lu R., Wada S., Zhang Y. (2020). Real-time penetration state monitoring using convolutional neural network for laser welding of tailor rolled blanks. J. Manuf. Syst..

[34] Kos M., Arko E., Kosler H., Jezeršek M. (2019). Remote laser welding with in-line adaptive 3D seam tracking. Int. J. Adv. Manuf. Technol..

[35] Chollet F. Keras. https://github.com/keras-team/keras.

[36] Ma N., Zhang X., Zheng H.-T., Sun J., Ferrari V., Hebert M., Sminchisescu C., Weiss Y. (2018). ShuffleNet V2: Practical Guidelines for Efficient CNN Architecture Design. Proceedings of the Computer Vision—ECCV 2018.

[37] Simard P.Y., Steinkraus D., Platt J.C. Best practices for convolutional neural networks applied to visual document analysis. Proceedings of the Seventh International Conference on Document Analysis and Recognition.

[38] Selvaraju R.R., Cogswell M., Das A., Vedantam R., Parikh D., Batra D. Grad-CAM: Visual Explanations from Deep Networks via Gradient-Based Localization. Proceedings of the 2017 IEEE International Conference on Computer Vision (ICCV).

